# Fatty Acid Metabolism Rewires Glioblastoma Progression and Treg-Mediated Immune Resistance

**DOI:** 10.3390/cancers18162573

**Published:** 2026-08-11

**Authors:** Nowreen Islam Chowdhury, Hebatollah Ewida, Mahmoud Salama Ahmed, Heidi Villalba

**Affiliations:** 1 School of Veterinary Medicine, Texas Tech University, Amarillo, TX 79106, USA; nowchowd@ttu.edu; 2 Department of Pharmaceutical Sciences, Jerry H. Hodge School of Pharmacy, Texas Tech University Health Sciences Center, Amarillo, TX 79106, USA; heba.ewida@ttuhsc.edu (H.E.); mahmoudsalama.ahmed@ttuhsc.edu (M.S.A.); 3 Brain Drug Discovery Center, Texas Tech University Health Sciences Center, Amarillo, TX 79106, USA

**Keywords:** glioblastoma, fatty acid oxidation, regulatory T cells, cancer metabolism, metabolic reprogramming, ICIs, lipid metabolism, tumor microenvironment

## Abstract

Glioblastoma (GBM) is one of the most aggressive and treatment-resistant cancers, shaped by a tumor microenvironment (TME) that is both metabolically demanding and strongly immunosuppressive. GBM relies heavily on fatty acid (FA) metabolism to sustain growth of rapidly dividing tumor cells and survive metabolic stress. GBM cells enhance lipid uptake, activate sterol regulatory element-binding protein 1 (SREBP-1)-driven lipogenesis, store excess lipids in droplets to prevent toxicity, and depend on fatty acid oxidation (FAO) to generate adenosine triphosphate (ATP) and maintain redox balance, particularly under nutrient-limited conditions. GBM TME is also consistently enriched with regulatory T cells (Tregs), which maintain suppressive activity despite the nutrient restrictions that impair effector T cells (Teffs). In hypoxia and nutrient limitation within the TME, Tregs can adapt by using FAO, lactate oxidation, and OXPHOS, supported by forkhead box P3 (Foxp3)-dependent metabolic programming, cluster of differentiation 36 (CD36)-mediated FA uptake, and hypoxia-related signals. At the same time, programmed cell death protein 1 (PD-1)/programmed death-ligand 1 (PD-L1) signaling reduces glycolytic activity in Teffs and contributes to metabolic dysfunction, while also supporting the stability of oxidative metabolism in Tregs. Evidence from pre-clinical and clinical studies suggests a possible association between Treg enrichment in GBM and reduced responsiveness to immune checkpoint inhibitors (ICIs), although this relationship is not yet fully defined. Overall, current findings point to FA metabolism as a shared metabolic axis that supports both tumor progression and Treg-mediated immune resistance. Targeting lipid-driven pathways may offer an opportunity to disrupt these advantages and improve the effectiveness of existing immunotherapies for GBM.

## Simple Summary

Glioblastoma (GBM) is the most lethal primary brain cancer, defined by its infiltrative nature and resistance to traditional therapies, including surgery, radiation therapy, and chemotherapy. GBM shows significant metabolic plasticity to adapt within the nutrient-limited or hypoxic tumor microenvironment (TME) to generate sufficient energy. Recent research shows that GBM cells rely heavily on fatty acid (FA) metabolism to support their growth, survival, and therapy resistance. Similarly, regulatory T cells (Tregs), key drivers of immune suppression in GBM, can also utilize FA as fuel, enabling them to maintain their suppressive activity within the tumor microenvironment. Evidence suggests that enrichment of Tregs within the GBM microenvironment may contribute to the limited success of immune checkpoint inhibitors (ICIs) in GBM. This review summarizes current knowledge of FA metabolism in GBM and Tregs and discusses emerging metabolic therapies. Targeting lipid metabolism may offer a promising strategy to simultaneously disrupt tumor growth and reduce immune suppression in GBM.

## 1. Introduction

Glioblastoma (GBM) is the most common and aggressive form of primary malignant brain tumor in adults, characterized by high mortality. Previously, gliomas were classified using the World Health Organization (WHO) four-tier grading system (grades I–IV), which was based entirely on histopathological features [1]. Grade I (pilocytic astrocytoma) was considered biologically indolent. Grade II (diffuse astrocytomas) showed infiltrative growth with low mitotic activity. Grade III (anaplastic astrocytomas) demonstrated increased cellularity and mitoses, showing a more aggressive clinical course. GBM represented grade IV, the highest and most malignant stage, defined by necrosis and microvascular proliferation. However, this framework changed substantially with the 2021 WHO Classification of Tumors of the Central Nervous System, which shifted from a purely morphology-based system to an integrated molecular histological approach. GBM is now defined as an adult-type, IDH1/2-wildtype diffuse astrocytic tumor, classified as central nervous system (CNS) WHO grade 4 [2]. In contrast, most adult WHO grade 3 astrocytomas are characterized by IDH1/2 mutations. GBM can be diagnosed not only by histological hallmarks but also by molecular features such as telomerase reverse transcriptase (TERT) promoter mutation, epidermal growth factor receptor (EGFR) amplification or combined chromosome 7 gain and chromosome 10 loss (+7/−10) [2]. To date, the gold standard therapy for GBM is surgical resection followed by radiotherapy (RT) and temozolomide (TMZ). Regardless of the intense treatment approaches, recurrence is inevitable in GBM with a median survival duration of 15–18 months, while only fewer than 5% of patients live beyond five years [3,4]. Several features of GBM contribute to these poor outcomes, including the tumor’s hypoxic microenvironment, marked intertumoral and intratumoral heterogeneity and limited permeability of the blood–brain barrier (BBB) restricting therapeutics delivery. Gene-expression studies classify GBM into three subtypes, namely proneural, classical, and mesenchymal [5]. Among these subtypes mesenchymal tumors show particularly aggressive behavior and resistance to therapy [6].

Historically, cancer metabolism has largely been interpreted through a glucose-centered framework, with the assumption that tumor cells primarily depend on glycolysis for their energy needs [7]. However, emerging evidence shows that GBM extends far beyond this glycolytic paradigm. Multiple studies have demonstrated that GBM relies on fatty acid (FA) metabolism, including FA uptake and fatty acid oxidation (FAO) as a major metabolic axis supporting tumor growth, survival, and redox balance [8,9]. This lipid-based pathway also generates bioactive lipid metabolites that shape the tumor immune landscape [9,10]. Clinical imaging and metabolic flux analyses further indicate that a substantial fraction of acetyl-CoA in human gliomas does not originate from glucose, suggesting that alternative metabolic substrates contribute significantly to tumor bioenergetics [11,12]. These insights have raised an interest in targeting FA metabolism to interfere with FA-driven energy production and disrupt the metabolic flexibility that helps GBM survive.

Another hallmark of GBM is the immunosuppressive tumor microenvironment (TME). The altered metabolic activity within the TME can facilitate the immunosuppressive characteristic of GBM. Regulatory T cells (Tregs) are major contributors to tumor-associated immunosuppression, reducing antitumor immunity and limiting the effectiveness of immune responses against GBM [13]. Interestingly, glioma patients commonly exhibit systemic T cell deficits and a marked enrichment of intratumor Tregs [14,15]. Beyond this numeric enrichment, metabolic constraints in the GBM TME differentially affect effector T cells (Teffs) and Tregs. The conventional Teffs rely heavily on glycolysis; therefore these cells become functionally impaired under glucose restriction in the TME. In contrast, forkhead box P3 (Foxp3^+^) Tregs are able to leverage mitochondrial oxidative pathways, including FAO, to support survival in the nutrient-limited, lactate-rich TME [10,16,17]. Studies in tumor models further show that lactic acid and hypoxia foster oxidative phosphorylation (OXPHOS)-driven highly immunosuppressive Treg phenotypes. In GBM, hypoxia-inducible factor-1α (HIF-1α) signaling has also been linked to a switch towards oxidative, immunosuppressive Tregs within the TME [16,18,19]. In GBM settings, Tregs continue to expand as the tumor grows, and they remain strongly suppressive even when immune checkpoint inhibitors (ICIs), including programmed cell death protein 1 (PD-1) inhibitors, are used to lift immune ‘brakes’ on T cells [20,21]. Although the mechanisms enabling Treg metabolic resilience have been described largely based on T cell biology, these principles are also relevant to GBM. For example, Foxp3-driven FAO and mitochondrial respiration in Tregs maintain activity in glucose-poor, lactate-rich conditions that impair Teffs [10]. This metabolic asymmetry directs a focus in targeting FA metabolism-linked pathways to weaken Treg fitness within the GBM microenvironment. Targeting these pathways may alleviate Treg-mediated checkpoint resistance and restore sensitivity to PD-1/PD-L1-based immunotherapy.

In this review, we focus on the dual roles of FA metabolism in GBM biology and Treg-mediated immunosuppression. We synthesize GBM-specific evidence where available and integrate broader immunometabolic studies to contextualize mechanisms that are not yet well established in gliomas. Finally, we explore whether therapeutically targeting lipid-driven pathways in both tumor cells and Tregs may offer a rational strategy to disrupt the immunosuppressive microenvironment and improve responsiveness to ICIs.

## 2. Fatty Acid Metabolism in GBM

### 2.1. Shifting Beyond the Warburg Paradigm

Previously, the Warburg effect dominated the interpretation of cancer metabolism [7]; however, recent studies have greatly broadened this perspective to further explore the adaptive metabolic reprogramming of GBM tumor to sustain growth [22,23]. Rapidly proliferating cancer cells require new cellular components and energy, where the tumor utilizes lipids for both anabolic and catabolic processes. FA are the building blocks of all lipids and serve as a primer for the synthesis of other lipids like glycerolipids, glycerophospholipids, sphingolipids, sterols, and saccharolipids [24]. Anabolic processes of tumors utilize lipids by directing them towards the synthesis of new cellular structures such as plasma membranes, organelle membranes, lipid droplets, and other macromolecular components required for biomass expansion [8,25]. Kant and colleagues added another layer of understanding by showing that GBM uses FAO in both nutrient-rich and-restricted settings. In nutrient-rich environments, FAO-derived β-hydroxybutyrate activates the receptor GPR109A to promote tumor cell proliferation. On the other hand, in nutrient-restricted settings, this same pathway shifts toward a catabolic role, where FAs undergo mitochondrial β-oxidation to generate ATP and maintain cellular survival [8].

As glial cells are highly glycolytic in nature; gliomas were also expected to follow a similar pattern. However, evidence from patient-derived imaging broadens this view of glioma metabolism including recurrent GBM. In 2002 Belohlávek and colleagues reported that 35–40% of recurrent gliomas could not be detected by measuring glucose uptake on fluorodeoxyglucose positron emission tomography (FDG-PET), even though they can still be seen on contrast magnetic resonance imaging (MRI) [11]. In addition, it was found that less than half of the acetyl-CoA in gliomas comes from blood-borne glucose, suggesting these tumors may use alternative fuels for their survival in the TME [12].

### 2.2. Lipid Uptake, SREBP-1 Signaling, and Enhanced Lipogenesis in GBM

GBM cells exhibit a strong dependency on lipid metabolism [8,22,23]. They actively import cholesterol through the low-density lipoprotein receptor (LDLR), whose expression is regulated by sterol regulatory element-binding protein-1 (SREBP-1). In GBM, SREBP-1 expression is substantially upregulated, promoting de novo FA biosynthesis and supporting rapid cell growth [26,27,28,29,30]. Under normal metabolic conditions, adenosine monophosphate-activated protein kinase (AMPK)-mediated phosphorylation of SREBP-1 suppresses its activity, thereby limiting lipid synthesis and tumor proliferation. AMPK also phosphorylates acetyl-CoA carboxylase (ACC), inhibiting FA synthesis [28,31]. However, GBM cells frequently maintain lipogenic activity even under energy stress, suggesting that the pathways normally suppressed by AMPK are rewired to support tumor growth [28,29,32]. Additionally, hyperactivation of the phosphoinositide 3-kinase (PI3K)/protein kinase B (AKT) pathway enhances SREBP-1 expression and transcription of genes involved in FA and cholesterol metabolism [33,34]. Mutations in EGFR further amplify this pathway, reinforcing GBM cell growth and survival through SREBP-1-mediated lipogenesis [29,30,32]. This aligns with broader evidence describing SREBP-dependent control of lipid synthesis in cancer cells [32].

Under physiological conditions, excess intracellular cholesterol suppresses SREBP-1 activation; however, studies show that GBM cells evade this sterol-mediated feedback mechanism and maintain SREBP-1 signaling [29,35,36]. Elevated levels of free FAs and cholesterol can induce endoplasmic-reticulum (ER) stress and trigger lipotoxic cell death [37,38,39]. GBM cells appear to evade this toxicity by converting surplus cholesterol into cholesteryl esters (CEs) and storing them within lipid droplets (LDs), which are highly abundant in human GBM tumor tissues but largely absent in normal brain [35]. Electron-microscopy and fluorescence-imaging studies have confirmed the presence of abundant lipid droplets in GBM patient tumors. Together, these observations support the idea that excess FAs and cholesterol are sequestered into lipid droplets, buffering free-lipid accumulation and reducing ER-stress-associated lipotoxicity [35].

Consistent with sustained lipogenesis, genetic suppression of acetyl-CoA carboxylase 1 (ACC1) and acetyl-CoA carboxylase 2 (ACC2) in EGFRvIII-driven GBM cells reduces de novo FA synthesis, induces apoptotic cell death, and impairs tumor growth, demonstrating a dependence on continued lipid anabolism [40]. Newly synthesized saturated FAs are desaturated to preserve membrane fluidity and ER homeostasis. This is a process mediated by stearoyl-CoA desaturase (SCD).

In GBM cells and glioma stem-like populations, genetic or pharmacologic inhibition of SCD disrupts ER integrity, reduces stem-cell frequency, and suppresses tumor growth in vivo, indicating that lipid desaturation is essential for tumor maintenance rather than merely increasing lipid abundance [41]. At the level of lipid-droplet biogenesis, GBM cells regulate neutral-lipid storage through diacylglycerol acyltransferase-1 (DGAT1)-dependent triglyceride synthesis and sterol O-acyltransferase-1 (SOAT1)-mediated cholesterol esterification. Experimental inhibition of SOAT1 blocks cholesterol-ester formation, destabilizes lipid-droplet stores, suppresses SREBP-1 activity, and markedly reduces GBM growth in xenograft models [35]. Together, these findings establish lipid synthesis, desaturation, and storage as coordinated, protective processes that enable GBM cells to tolerate metabolic stress. In parallel, DGAT1 inhibition disrupts triglyceride storage, leading to excessive FAO, reactive-oxygen-species accumulation, and tumor cell death [26].

In a cohort of 80 glioma patients (46 grade II, 34 grade IV), GBM samples exhibited higher carnitine palmitoyltransferase-1A (CPT1A) expression, suggesting a metabolic shift toward FAO in high-grade gliomas [42]. CPT1A regulates the entry of long-chain FAs into mitochondria, and FAO supplies tumor cells with both ATP and nicotinamide adenine dinucleotide phosphate (NADPH). Pharmacologic inhibition of FAO with etomoxir reduces NADPH levels, increases reactive-oxygen-species accumulation, and triggers GBM cell death [9].

This metabolic shift towards FAO is also an important feature of recurrent GBM. GBM almost invariably recurs and patients may experience multiple episodes of recurrence throughout disease progression [43]. Recurrent GBMs exhibit biological changes compared to matched primary tumors, including clonal evolution, acquisition of new genetic alterations, transcriptional subtype switching demonstrating substantial molecular evolution during disease progression [44]. Metabolic alterations during GBM recurrence have also been investigated using paired primary and recurrent GBM specimens [45]. Tsai and colleagues conducted transcriptomic and metabolomic analyses of paired primary and recurrent GBM specimens, which identified reprogramming of arachidonate metabolism in recurrent tumors. The authors then used TMZ-sensitive and TMZ-resistant GBM cell models to demonstrate that this metabolic reprogramming enhanced mitochondrial metabolism, including FAO and TCA cycle activity. Furthermore, inhibition of prostaglandin E2 (PGE2) signaling or knockdown of CPT1A attenuated FAO and increased TMZ sensitivity, suggesting that FAO contributes to the metabolic adaptations associated with TMZ resistance [45].

**Figure 1:**
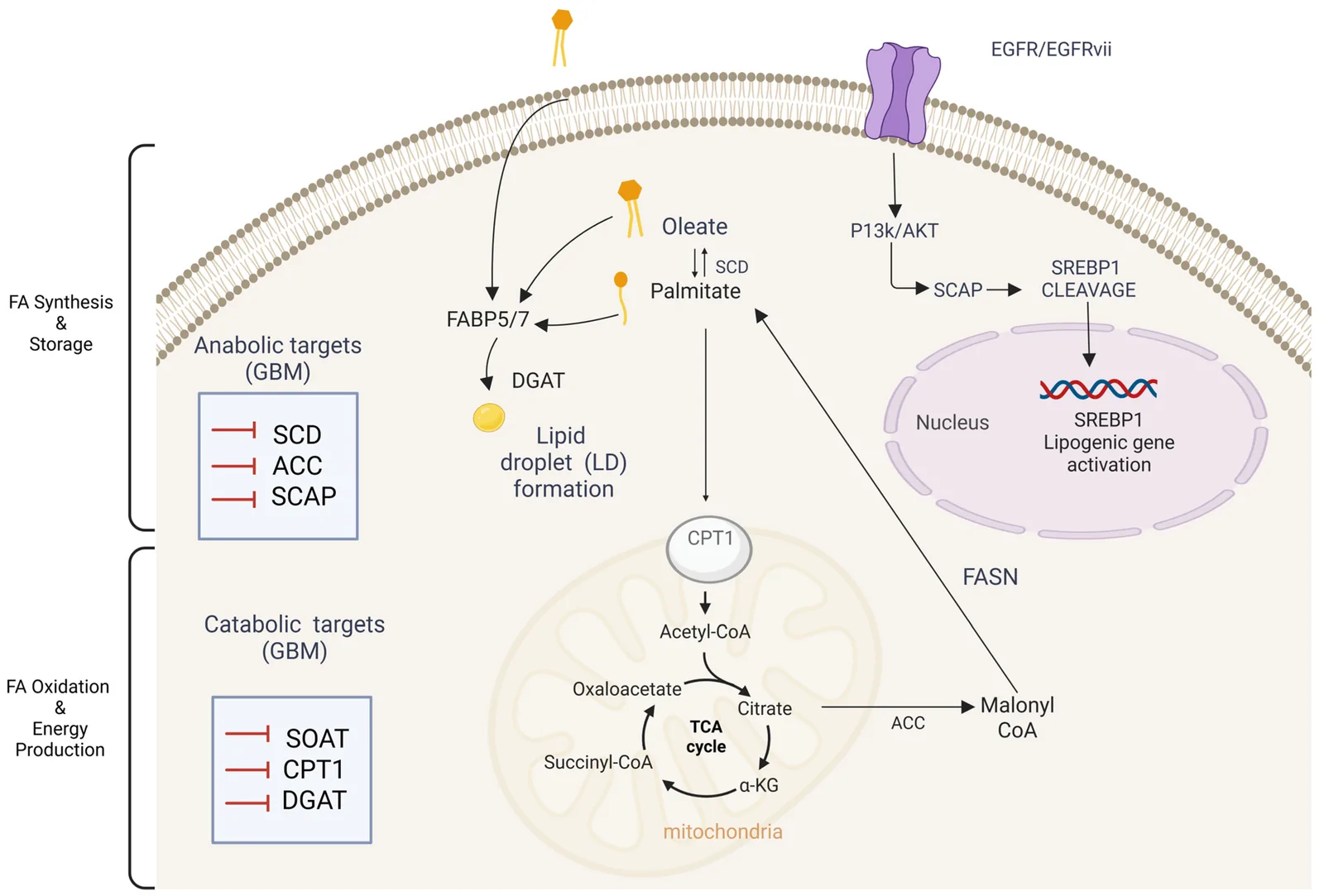
Fatty acid anabolism and catabolism in GBM. Abbreviations: FA: fatty acid; GBM: glioblastoma; EGFR: epidermal growth factor receptor; EGFRvIII: epidermal growth factor receptor variant III; PI3K: phosphoinositide 3-kinase; AKT: protein kinase B; SCAP: SREBP cleavage-activating protein; SREBP1: sterol regulatory element-binding protein 1; FABP5/7: fatty acid-binding proteins 5 and 7; DGAT: diacylglycerol O-acyltransferase; LD: lipid droplet; SCD: stearoyl-CoA desaturase; CPT1A: carnitine palmitoyltransferase 1; CoA: coenzyme A; TCA: tricarboxylic acid; α-KG: alpha-ketoglutarate (2-oxoglutarate); ACC: acetyl-CoA carboxylase; FASN: fatty acid synthase; SOAT: sterol O-acyltransferase.

GBM reprograms FA metabolism to support tumor growth and metabolic adaptation through coordinated anabolic and catabolic programs that govern FA synthesis, modification, storage, and utilization (Figure 1). As depicted schematically, FA anabolism is driven by oncogenic signaling pathways, particularly EGFR/PI3K/AKT, which activate the SREBP cleavage-activating protein (SCAP)–SREBP-1 axis to induce transcription of lipogenic genes and promote de novo FA synthesis. Experimental studies demonstrate that disruption of this regulatory axis suppresses GBM growth and sensitizes tumors to metabolic stress [29]. Downstream, ACC catalyzes the rate-limiting step in FA synthesis by generating malonyl-CoA, and ACC inhibition reduces proliferation and induces apoptosis in EGFRvIII-positive GBM models [40,46]. Newly synthesized saturated FAs are further processed by stearoyl-CoA desaturase (SCD) to generate monounsaturated FAs required for membrane homeostasis, ER integrity, and lipid storage. Genetic or pharmacologic SCD inhibition disrupts ER homeostasis, reduces glioma stem-cell frequency, and suppresses tumor growth in vivo [41,47]. As illustrated, excess or newly synthesized FAs can be sequestered into lipid droplets, providing a buffering reservoir that limits lipotoxicity and regulates FA availability. Under conditions of nutrient limitation, hypoxia, or therapeutic stress, GBM cells can mobilize FAs from this pool toward mitochondrial β-oxidation. This adaptive catabolic program depends on CPT1A, and multiple studies show that inhibition of FAO impairs GBM survival and metabolic plasticity, despite limitations of available CPT1A inhibitors [8,22]. This lipid routing between storage and oxidation is further controlled by lipid-droplet regulatory enzymes, including diacylglycerol acyltransferase-1 (DGAT1), which mediates triglyceride synthesis, and sterol O-acyltransferase-1 (SOAT1), which catalyzes cholesterol esterification; inhibition of either DGAT1 or SOAT1 disrupts lipid-droplet homeostasis, increases oxidative stress, and suppresses GBM growth in pre-clinical models [35]. Targets highlighted in the schematic represent metabolic control nodes that have been experimentally perturbed in GBM to disrupt FA anabolic or catabolic programs, rather than implying exclusive pathway specificity or direct clinical tractability. The figure was created by the authors in https://BioRender.com and the conceptual framework is adapted from Miska and Chandel [48].

### 2.3. FAO-Driven Radioresistance in GBM

Although current gold standard treatment of GBM includes radiotherapy following surgical resection, some aggressive tumor cells often survive radiotherapy and develop more aggressive and therapy-refractory features. These recurrent and radioresistant (RR) GBM cells reprogram their metabolism from glycolysis toward FAO, characterized by increased lipid utilization, and enhanced mitochondrial respiration [49,50,51,52]. This metabolic rewiring not only provides higher energy output but also drives immune escape by inducing CD47 expression, a key “don’t eat me” signal [53]. Immunohistochemical analysis of 46 paired primary and recurrent high-grade glioma specimens demonstrated increased expression of the FAO enzymes CPT1A, CPT2, and ACAD9 in 86.96%, 84.78%, and 76.09% of recurrent tumors, respectively, together with increased CD47 expression in 82.6% of cases [54]. The elevated CPT1A, CPT2, and CD47 expression were each associated with reduced overall survival. In complementary experimental studies, RR GBM models exhibited enhanced FAO and increased CD47 expression compared with their parental counterparts [54]. Although radiation increases blood–brain barrier (BBB) permeability and enhances the delivery of anti-CD47 antibodies to the tumor, the elevated CD47 expression in recurrent and RR GBM may limit their intratumoral distribution through rapid antibody binding at the tumor surface, presenting an additional therapeutic challenge [54]. These findings indicate that the FAO–CD47 axis contributes to the metabolic and immune adaptations of RR GBM, providing a rationale for therapeutically targeting both pathways.

## 3. Immunosuppressive Regulatory T Cells in GBM

**Figure 2:**
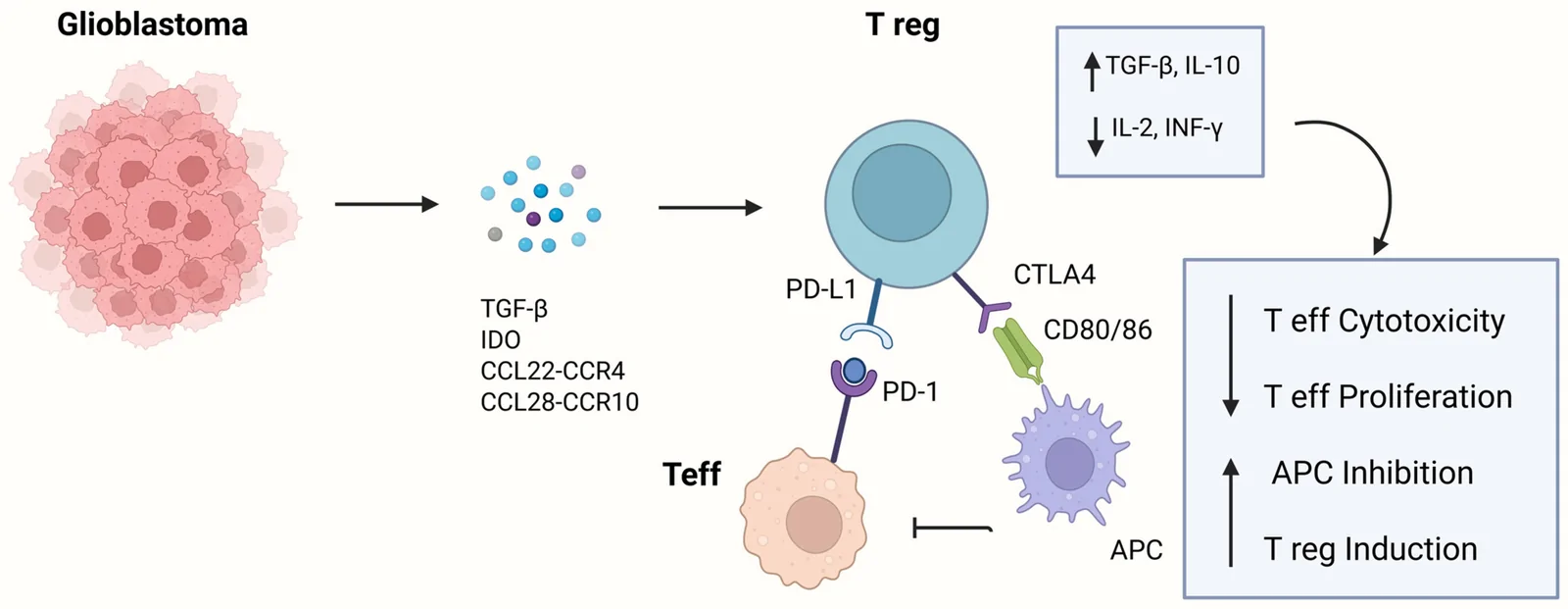
Regulatory T cell-mediated immunosuppression in glioblastoma. Abbreviations: Treg: regulatory T cell; Teff: effector T cell; APC: antigen-presenting cell; TGF-β: transforming growth factor-beta; IDO: indoleamine 2,3-dioxygenase; CCL22: C-C motif chemokine ligand 22; CCR4: C-C chemokine receptor 4; CCL28: C-C motif chemokine ligand 28; CCR10: C-C chemokine receptor 10; PD-L1: programmed death-ligand 1; PD-1: programmed cell death protein 1; CTLA-4: cytotoxic T-lymphocyte-associated protein 4; CD80/86: cluster of differentiation 80/86; IL-10: interleukin-10; IL-2: interleukin-2; IFN-γ: interferon-gamma.

One of the hallmark findings in GBM patients is profound immune suppression characterized by a reduced number of circulating cluster of differentiation 4 (CD4^+^) T cells, accompanied by an increased proportion of Tregs (Figure 2) [15]. Tumor-derived factors such as chemokine C-C ligand 2 (CCL2) promote the recruitment of Tregs into the GBM microenvironment [55,56]. Within the TME, Treg function can be further amplified by indoleamine 2,3-dioxygenase (IDO), an enzyme frequently overexpressed in gliomas and other cancers. Additionally, Glioma-derived chemokines also promote the preferential recruitment of Tregs into the TME through chemokine receptor type 4 (CCR4)-dependent mechanisms. Although C-C motif chemokine ligand 22 (CCL22) has been implicated in this process, available evidence suggests that the CCR4-mediated pathway supports Treg accumulation in GBM as part of a broader network of tumor-derived signals, rather than acting as a single dominant recruitment pathway [56].

Tregs can arise either as naturally occurring Tregs (nTregs) from the thymus or as induced Tregs (iTregs) in response to antigens in tolerogenic settings. As illustrated in Figure 2, Tregs suppress immune activity through multiple mechanisms including release of inhibitory cytokines such as transforming growth factor-β (TGF-β) and interleukin-10 (IL-10) and exerting direct cell-to-cell inhibition [57]. Tregs constantly express cytotoxic T-lymphocyte-associated protein 4 (CTLA-4) that binds with high affinity to the costimulatory molecules CD80 and CD86 on antigen-presenting cells (APCs). This interaction prevents APCs from engaging with CD28 on Teffs, thereby inhibiting the costimulatory signaling required for their complete activation. Tregs also express PD-L1, which engages with PD-1 on Teffs, promoting apoptosis and functional exhaustion resulting in immunosuppressive TME [58,59]. The immunosuppressive activity and persistence of these Tregs within the TME is increasingly recognized to depend on distinct metabolic adaptation, specifically FA metabolism [60].

GBM cells secrete immunomodulatory factors like TGF-β, indoleamine 2,3-dioxygenase (IDO), and the chemokines CCL22 and CCL28, which recruit and promote the differentiation of Tregs via CCR4-dependent signaling [55,56,59]. Within the TME, Tregs suppress antitumor immune responses through multiple mechanisms, including expression of immune checkpoint molecules and immunosuppressive cytokines [13,57,59]. Tregs inhibit ICI function via PD-1/PD-L1 signaling and attenuate APC activation through CTLA-4-mediated engagement of CD80/CD86 [13,57,58]. Functionally, Treg activity is associated with increased production of TGF-β and IL-10, reduced effector cytokines such as interleukin-2 (IL-2) and interferon-γ (IFN-γ), decreased Teff cytotoxicity and proliferation, enhanced inhibition of APC activation, and reinforcement of an immunosuppressive TME that favors Treg induction and persistence [13,15,20,21,59]. The figure was created by the authors in https://BioRender.com and the conceptual framework is adapted from Grabowski and colleagues [61].

## 4. Mitochondrial and Fatty Acid-Driven Metabolic Pathways in Tregs

The TME is characterized by hypoxia and intense nutrient competition that can impair the function of glycolysis-dependent Teffs. However, Tregs are less vulnerable to glucose restriction because they rely more strongly on mitochondrial oxidative metabolism. A central determinant of this metabolic preference is Foxp3. Foxp3 is a transcription factor that is crucial for the development and function of Tregs. It establishes a transcriptional profile that enables efficient use of long-chain FAs as fuel in the TME [10]. Foxp3 induces enzymes involved in FA activation (long-chain acyl-CoA synthetase family members), mitochondrial import (CPT1A), and β-oxidation within the mitochondrial matrix (acyl-CoA dehydrogenase and enoyl-CoA hydratase proteins), creating a coordinated FAO module. Functionally, Foxp3^+^ cells show enhanced palmitate-supported oxygen consumption, and this FA metabolism-linked respiration is impaired when CPT1A is pharmacologically inhibited with etomoxir [10]. Also, Foxp3-dependent FAO provides a survival advantage in lipid-rich environments. Foxp3^+^ cells resist lipotoxicity triggered by long-chain FAs, whereas Foxp3^−^ (conventional T cells) cells are highly susceptible to this. This indicates that Foxp3 shapes mitochondrial metabolism at multiple levels including enhancing FA transport, β-oxidation enzymes, and respiratory-chain activity to generate a metabolic state in which FAO functions both as energy source and buffer against lipotoxic stress [10]. However, the extent to which Tregs rely on FAO remains debated, as some studies report preserved Treg suppressive function despite FAO inhibition, emphasizing that Tregs can shift between FAO, glycolysis, and lactate oxidation depending on the metabolic landscape [62].

In GBM, tumor cells convert pyruvate to lactate through lactate dehydrogenase (LDH), producing a lactate-rich and glucose-poor microenvironment [63]. Tregs in this setting express the monocarboxylate transporters 1 (MCT1), enabling uptake of lactate and lactic acid [18,64]. Once internalized, lactate is reconverted into pyruvate by LDH, entering the tricarboxylic-acid (TCA) cycle to fuel mitochondrial respiration [18]. This lactate-supported oxidative metabolism reduces reliance on glycolysis and promotes an OXPHOS-driven phenotype to preserve Treg suppressive activity even when glucose is scarce. Angelin and colleagues have shown that Tregs support their survival in an environment enriched with lactic acid and depleted of glucose [16]. Thus, within glioma, Tregs use both FAO-mediated OXPHOS and lactate oxidation to sustain mitochondrial function under nutrient stress.

Hypoxia further modulates Treg behavior through HIF-1α. Tumor or immune cell expression of HIF-1α can increase molecules such as CCL20, TGF-β, and vascular endothelial growth factor A (VEGF-A), which have been associated with Treg recruitment to the TME [65,66]. The influence of HIF-1α on mature Tregs is strongly context dependent. Interestingly, HIF-1α has been reported to exhibit both pro and anti-regulatory effects on Treg biology, indicating that its functional impact of Treg varies with metabolic and microenvironmental conditions under which Tregs are examined. Early T cell metabolism studies show that HIF-1α can enhance Foxp3 expression and promote Treg differentiation under hypoxic conditions [67]. In contrast, other work demonstrates that sustained HIF-1α-driven glycolysis can impair Treg suppressive activity by diverting pyruvate away from mitochondrial oxidation and reinforcing a glycolytic metabolic bias [19,68]. HIF-1α has also been reported to interact with Foxp3 and contribute to its degradation through Prolyl Hydroxylase Domain (PHD)–Von Hippel–Lindau (VHL)-mediated ubiquitination degradation pathways [69]. Consistent with this complexity, Tregs lacking HIF-1α show altered responses to metabolic disruption, suggesting functional crosstalk between HIF-1α signaling and lipid metabolism [19].

Additional FAO-related pathways support Treg fitness in tumors. Tregs can take up long-chain FAs through cluster of differentiation 36 (CD36) and inhibiting CD36-mediated lipid import reduces intratumoral Treg accumulation in mouse melanoma without causing systemic immune instability. CD36-mediated FA uptake can activate PPAR-β signaling, supporting mitochondrial respiration and contributing to Treg function [60,70,71]. Although this pathway has been characterized by other solid tumors, similar lipid-driven mechanisms are likely relevant in GBM due to its lipid-rich metabolic landscape. AMPK promotes FAO by activating CPT1A, and pharmacologic inhibition of CPT1A with etomoxir reduces Treg proliferation by limiting FA transport into mitochondria [17]. Liver Kinase B1 (LKB1), an upstream regulator of AMPK, supports oxidative metabolism and prevents Tregs from deviating toward Th1 or Th17 phenotypes, and it also links to the mevalonate–cholesterol pathway that influences Treg stability [72].

Together, these findings suggest that Tregs possess metabolic flexibility that allows them to rely on FA metabolism and OXPHOS under glucose depletion conditions. By shaping a lipid-rich, lactate-rich, and glucose depleted tumor environment, GBM’s reliance on FA metabolism creates metabolic conditions in which Tregs maintain fitness while glycolysis-dependent ICIs become metabolically impaired.

## 5. PD-1 Signaling in Glioblastoma and Relevance to FAO-Linked T Cell Exhaustion

PD-1 signaling is strongly active within the GBM microenvironment. In GBM, tumor-infiltrating CD4^+^ and CD8^+^ T cells display high levels of PD-1 together with features of metabolic dysfunction and exhaustion [73,74,75]. Although PD-L1 is classically described as a ligand on tumor cells or APCs, one study reported that GBM-infiltrating CD4^+^ and CD8^+^ T cells can also express PD-L1 [76]. This unusual expression pattern introduces an additional layer of immunosuppression, as PD-L1 can bind CD80 (B7-1) and interfere with CD28 co-stimulation, resulting in exhausted T cell phenotype [77]. Phosphatase and Tensin Homolog (PTEN) loss is a frequent event in GBM that further enhances PD-L1 expression [74]. Although PD-L1 is expressed in most GBM cases, its prognostic impact has varied across studies and analytic platforms [75]. The discrepancy may have stemmed from differences in analytic platforms, where microarray-based data did not reveal survival differences, but RNA-Seq analyses aligned with immunohistochemical findings demonstrated a clear negative association.

While PD-1/PD-L1 rich immunosuppressive environment in GBM has been established, the downstream metabolic effects of PD-1 signaling have not yet been defined specifically in glioma. However, mechanistic studies in T cells provide important insight like that PD-1 engagement recruits Src homology region 2 domain-containing phosphatase-2 (SHP-2) to its immunoreceptor tyrosine-based inhibitory/switch motifs (ITIM/ITSM) and suppresses CD28/TCR-dependent signaling pathways, including Ras/MAPK and the PI3K–Akt–mammalian target of rapamycin (mTOR) [78,79,80]. This suppression reduces glycolysis and amino-acid uptake while promoting lipolysis and CPT1A-dependent FAO partly through induction of adipose triglyceride lipase (ATGL) [81]. This PD-1-driven metabolic shift increases reliance on polyunsaturated FAs. In oxidative tumor environments it can undergo peroxidation and impair T cell fitness. Oxidized lipids can be taken up through CD36 to drive CD8^+^ T cell dysfunction in tumor settings [82]. In contrast, Tregs are metabolically advantaged under these conditions, as PD-1 and TGF-β/SMAD3 signaling reinforce OXPHOS and FA-driven metabolic programs. This phenomenon stabilizes and enhances Treg suppressive function [83,84]. These findings suggest that the combination of a PD-1/PD-L1-dominated microenvironment and lipid-rich tumor conditions creates a metabolic context in which Teffs become exhausted while Tregs remain preferentially supported.

## 6. Treg-Mediated ICI Resistance in Glioblastoma

### 6.1. Barriers to Immune-Checkpoint Efficacy in GBM

ICIs are monoclonal antibodies that target inhibitory checkpoint molecules expressed on APCs and T lymphocytes, particularly PD-1 and CTLA-4 [85]. ICIs have shown remarkable efficacy in several malignancies by reversing T cell exhaustion and restoring cytotoxic activity [86]. A number of ICIs such as nivolumab and pembrolizumab (anti-PD-1), atezolizumab and durvalumab (anti-PD-L1), and ipilimumab (anti-CTLA-4) have been approved by the U.S. Food and Drug Administration (FDA) for use in cancers including Hodgkin lymphoma, head and neck squamous cell carcinoma, urothelial carcinoma, renal cell carcinoma, and microsatellite instability-high (MSI-high) colorectal cancer [87,88,89,90,91].

Developing and delivering effective treatments for GBM remains highly challenging due to several physiological constraints. Although occasional responses have been reported, randomized trials have not demonstrated a survival benefit of ICIs in recurrent GBM. In the phase III CheckMate-143 trial, nivolumab failed to improve overall survival compared with bevacizumab in recurrent GBM [92]. Similarly, in a randomized phase II study, pembrolizumab with or without bevacizumab did not improve outcomes in recurrent GBM [93]. GBM has a complex biology, and it grows in a diffuse, infiltrative manner, making complete removal difficult. Also, GBM shows significant heterogeneity, both within a single tumor and across different patients which makes it difficult to design a uniform treatment strategy across a group of patients [94].

Additionally, the brain is immune-privileged which means infiltration by the body’s circulating immune cells or drugs is restricted by the BBB [95]. Surprisingly, compared to healthy controls, GBM patients often exhibit T cell lymphopenia and reduced major histocompatibility complex class II (MHC II) expression on circulating monocytes [96]. In some cohorts, it was reported that treatment-naive GBM patients have severely suppressed immunity even before therapy begins. Studies have shown that these patients often have significantly reduced circulating T cell counts, sometimes reaching acquired immunodeficiency syndrome (AIDS) level CD4^+^ numbers [14]. Their spleens and other lymphoid organs are also smaller than normal, indicating a loss of immune cell reserves. This tumor-induced sequestration limits immune surveillance and contributes to systemic immunosuppression [14].

### 6.2. Treg Accumulation and Checkpoint-Mediated Immunosuppression

While the overall pool of Teffs is diminished, the GBM TME becomes enriched with Tregs [15]. This accumulation is promoted by immunosuppressive cytokines such as TGF-β, IL-10, and CCL2 secreted by glioma and myeloid cells [55,56]. TGF-β promotes the differentiation of a CD4^+^ T cells into FoxP3^+^ Tregs. These Tregs possess potent suppressive activity and can constitute up to 25% of tumor-infiltrating lymphocytes (TILs) in GBM [97]. Importantly, these Tregs rely on specialized metabolic programs, including FA metabolism, to maintain their persistence and immunosuppressive activity within the GBM microenvironment [19].

ICIs such as anti-PD-1 and anti-CTLA-4 antibodies were initially developed with a goal to reactivate exhausted Teffs and restore their ability to attack tumor cells. However, subsequent findings revealed that Tregs in the GBM microenvironment also express PD-1 and CTLA-4, often at higher levels than Teffs. Therefore, blocking PD-1 can unintentionally enhance the proliferation and suppressive function of Tregs [20,59,98].

### 6.3. Metabolic and Cytokine Mechanisms of Treg-Driven Checkpoint Resistance

Tregs play a persistent role in the failure of ICIs. Within the nutrient-poor and hypoxic TME, GBM-infiltrating Tregs display remarkable metabolic plasticity, enabling them to maintain suppressive function even under conditions causing Teff exhaustion. Specifically, Tregs adapt by upregulating the FA transporter CD36, a scavenger receptor for long-chain FAs and oxidized lipoproteins. This adaptation allows them to utilize lipid-derived energy rather than relying on glycolysis, which is impaired in the glucose deprived TME. CD36 activates peroxisome proliferator-activated receptor-β (PPAR-β) signaling, enhancing mitochondrial biogenesis, OXPHOS, and FAO, thereby maintaining ATP production and nicotinamide adenine dinucleotide (NAD^+^) homeostasis necessary for Treg survival and suppressive stability [60].

In 2021, Amoozgar and colleagues provided compelling mechanistic evidence that Tregs are a central driver of ICI failure in GBM [20]. They showed that murine GBM tumors are heavily infiltrated by Tregs [15,99] and their frequency rises in proportion to tumor burden [20]. These intratumoral Tregs adopt a strongly suppressive, anergic phenotype Helios^+^ and IL-10^+^ which becomes more pronounced as tumors enlarge and is further exacerbated by PD-1 blockade [20]. Their observation is consistent with clinical observations that PD-1 inhibitors show limited benefit in GBM [92,93]. Amoozgar and colleagues confirm that anti-PD-1 did not improve survival and did not enhance CD8^+^ T cell infiltration in their models. Rather PD-1 therapy intensifies Treg anergy and strengthens Treg-mediated immunosuppression instead of reversing it.

Beyond the functional reprogramming of Tregs, their phenotypic characteristics and mechanisms of immune suppression have also been investigated in GBM. Single-cell RNA-seq datasets of primary and recurrent GBM exhibit a GBM-specific Treg gene signature that correlates with (CGGA) cohorts [21]. Cytometry by time of flight (CyTOF) and flow cytometry indicate that most intratumoral CD4^+^, CD8^+^, and Treg populations in human GBM are PD-1^+^ KI67^−^, with Tregs forming a distinct CD25 high, CTLA-4 high subset [21]. CD4^+^Foxp3^+^ Tregs limit antitumor immunity through suppressive cytokines and metabolic competition [100]. Their high-affinity for IL-2 receptor (CD25) allows them to consume IL-2 that would generally support the proliferation of CD8^+^ and conventional CD4^+^ T cells [101]. In GBM, most of the intratumoral T cells exhibit a PD-1^+^KI67^−^ phenotype that is consistent with antigen experience but minimal proliferative activity. This low-proliferative profile aligns with the idea that CD25^+^ Tregs restrict IL-2 availability and thereby limit Teff activation.

To determine whether direct depletion of Tregs could overcome immune suppression, Galvez-Cancino and colleagues used a non-IL-2-blocking anti-CD25 antibody (anti-CD25NIB) which depletes Tregs while preserving IL-2 signaling. In the Nf1^−^/^−^Pten^−^/^−^EGFRvIII^+^ GBM model, Treg depletion enabled PD-1^+^KI67^−^ CD8^+^ T cells to enter the cell cycle and transition into a PD-1^+^KI67 high granzyme B (GZMB^+^) effector population, indicating restored proliferation and cytotoxic differentiation. Importantly, anti-PD-1 monotherapy had little effect in this model initially, but once Tregs were removed, anti-PD-1 produced substantially greater expansion of effector CD8^+^ T cells and improved survival. This result demonstrates that PD-1-based ICIs become effective when the Treg–IL-2 barrier is lifted. This effector transition required both IL-2 and CD8^+^ T cells, as IL-2 neutralization or CD8 depletion prevented KI67 induction, granzyme expression, and tumor control. Tregs promote checkpoint resistance in GBM by consuming IL-2 and maintaining intratumoral CD8^+^ T cells in a non-proliferative state [21]. Therefore, targeting Treg depletion can relieve this constraint and enhance responsiveness to PD-1 blockade.

In addition to antibody-mediated Treg depletion, low-dose cyclophosphamide has been proposed as an alternative approach to modulate Treg-mediated immunosuppression. Heylmann and colleagues demonstrated that human CD4^+^CD25^+^FoxP3^+^ Tregs are particularly sensitive to low-dose cyclophosphamide [102]. Consistent with this concept, metronomic cyclophosphamide has also been investigated as an immunomodulatory strategy in glioma. In an immunocompetent GL261 glioma model, metronomic cyclophosphamide induced sustained CD8^+^ T cell-dependent antitumor immunity and durable tumor regression [103]. This was also accompanied by reduced expression of FoxP3, a lineage-defining transcription factor of Tregs. FoxP3 expression was inversely correlated with cytotoxic Teff markers and increased after treatment cessation as cytotoxic activity declined [103]. These findings support further investigation of cyclophosphamide as an adjunct to immunotherapy; however, whether low-dose cyclophosphamide enhances immune checkpoint blockade through Treg modulation in GBM remains to be established.

## 7. Therapeutic Implication of Targeting Metabolism in GBM

The current first-line standard for newly diagnosed GBM patients is the Stupp regimen [104]. This regimen includes maximal safe surgical resection followed by RT with concurrent TMZ and subsequent adjuvant TMZ. In the landmark randomized phase III trial conducted by Stupp and colleagues, addition of TMZ to RT showed significant improvement in median overall survival from 12.1 to 14.6 months and also increased the 2-year survival rate from 10.4% to 26.5%, establishing this regimen as the therapeutic backbone for newly diagnosed GBM [104]. However, prolonged TMZ exposure alone has not consistently improved outcomes. For example, dose-dense adjuvant TMZ did not improve overall or progression-free survival compared with standard TMZ and greater toxicity was reported [105]. These findings suggest that improving therapeutic outcomes will likely require incorporation of mechanistically complementary agents rather than increasing the dose or duration of TMZ.

Targeting GBM metabolism may offer a complementary strategy for improving the efficacy of the Stupp regimen by exploiting metabolic adaptations induced by TMZ and RT. Parik and colleagues demonstrated that TMZ suppresses FA synthesis and desaturation in newly diagnosed GBM cells, whereas this metabolic response is attenuated in patient-derived recurrent GBM cells. The study reported pharmacological inhibition of SCD and fatty acid desaturase 2 (FADS2) increased saturated FA accumulation and synergistically enhanced TMZ-induced cell death, including in cells derived from recurrent tumors [106]. Similarly, De Martino and colleagues showed that RT induces a lipogenic adaptation characterized by accumulation of unsaturated fatty acids and lipid droplets [107]. This protects GBM cells from ER stress. Genetic or pharmacological inhibition of FASN showed increased apoptosis after irradiation, and combining FASN inhibition with focal RT prolonged the survival in GBM-bearing mice [107]. Together, these studies provide direct pre-clinical evidence that targeting FA synthesis and desaturation may sensitize GBM to both TMZ and RT and may therefore represent a rational adjunct to the Stupp regimen.

Beyond lipid metabolism, several other metabolic vulnerabilities have also been explored as therapeutic adjuncts to standard GBM treatment. Inhibition of the cystine–glutamate antiporter system Xc^−^ (xCT) with sulfasalazine enhanced TMZ-induced cytotoxicity in human GBM cells [108,109]. Similarly, the xCT inhibitor erastin has been reported to sensitize GBM cells to TMZ by promoting ferroptotic lipid peroxidation through inhibition of cystine uptake and depletion of intracellular glutathione [110]. In parallel, metformin has emerged as a clinically investigated metabolic modulator that may help provide a translational framework for integrating metabolism-targeted therapies with current treatment. In a phase I study of patients with newly diagnosed GBM who had completed concurrent RT/TMZ, metformin at doses up to 2250 mg/day was combined with maintenance TMZ without dose-limiting toxicities [111]. Another phase I study demonstrated that metformin-containing multidrug combinations could be used with post-radiation adjuvant TMZ [112]. Collectively, these findings suggest that metabolism-targeted therapies may represent promising adjuncts to standard RT and TMZ by targeting treatment-induced metabolic adaptations; however, their clinical utility remains to be established in prospective clinical trials.

Another glycolysis-targeting strategy investigated in GBM is 2-deoxy-D-glucose (2-DG), a glucose analog that is transported into cells through facilitative glucose transporters (GLUTs), including GLUT1 and GLUT3, where it is phosphorylated by hexokinase to inhibit glycolysis [113]. Zhang and colleagues reported that GBM cells actively take up 2-DG in a GLUT1-dependent manner, and disruption of GLUT1 membrane localization significantly reduces 2-DG uptake, glycolysis, and tumorigenic potential [114]. Although 2-DG does not directly target lipid metabolism, its inclusion is supported by the close metabolic crosstalk between glycolysis and lipid metabolism. Wang and colleagues demonstrated that simultaneous inhibition of glycolysis with 2-DG and autophagy-mediated lipid catabolism or FAO produced greater antitumor effects than targeting either pathway alone [115]. Early Phase I/II clinical studies demonstrated that combining 2-DG with radiotherapy in patients with high-grade glioma was feasible and generally well tolerated [116]. In pre-clinical GBM models, 2-DG also enhanced the cytotoxic effects of alkylating chemotherapy by suppressing glycolysis, increasing oxidative and ER stress, and promoting apoptosis [117]. However, subsequent clinical translation in GBM has been limited, and conflicting pre-clinical findings, including reports of increased glioblastoma cell migration and invasion under certain conditions, indicate that additional optimization is required before 2-DG can be considered a therapeutic option for GBM [118]. Nevertheless, continued clinical evaluation of 2-DG in non-oncologic indications highlights ongoing interest in its pharmacological potential, which may inform the future development of improved glycolytic inhibitors [119].

**Figure 3:**
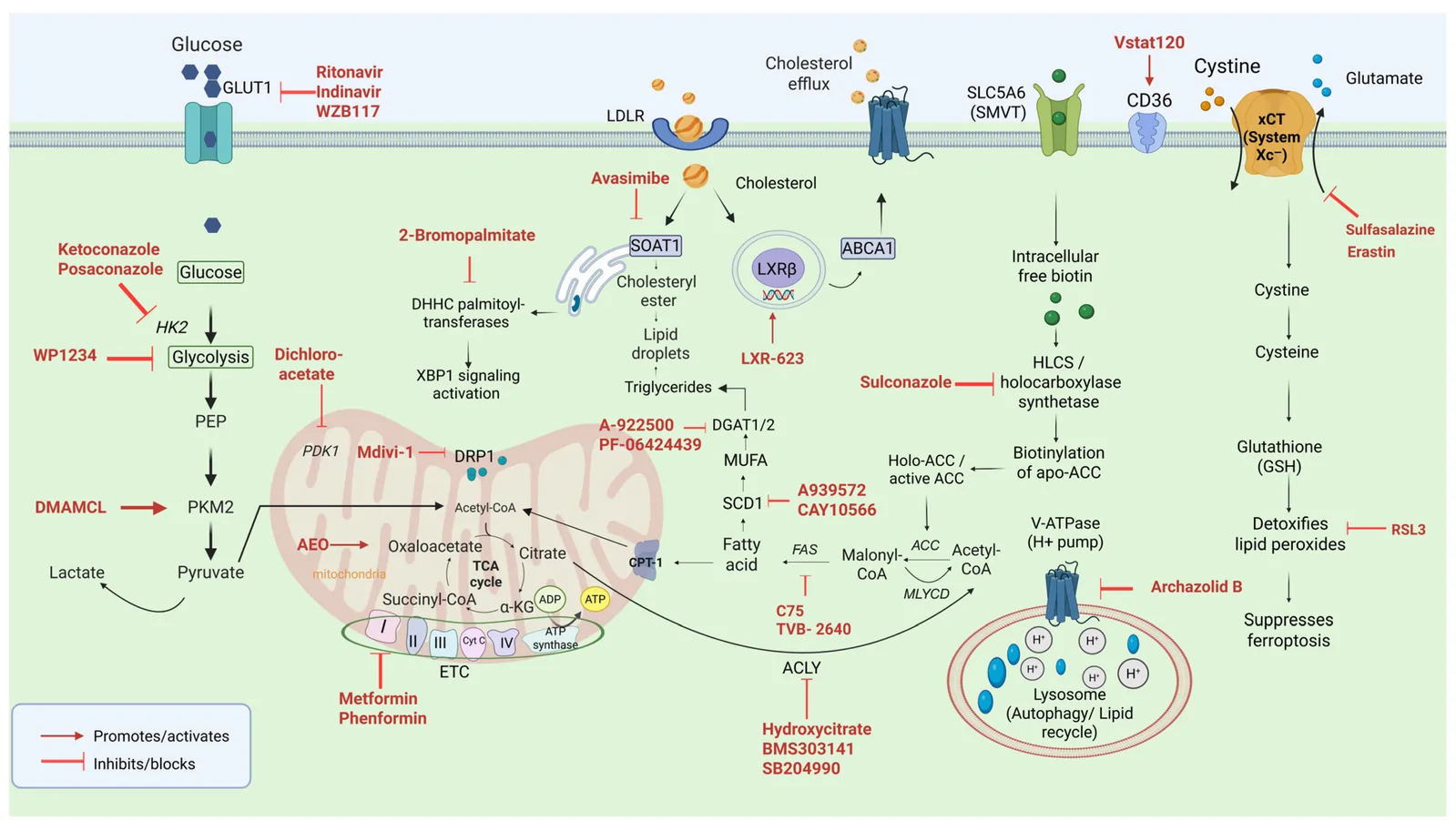
Schematic overview of metabolic pathways targeting therapeutics in GBM.

To date, experimental work has identified multiple metabolic pathways essential to GBM survival. These metabolic vulnerabilities and their corresponding therapeutic targets are summarized in Figure 3.

This figure summarizes metabolic pathways that have been investigated as potential therapeutic vulnerabilities in GBM including targeting glucose uptake and glycolysis (GLUT1, HK2, and PKM2), mitochondrial metabolism and OXPHOS (PDK1, oxaloacetate metabolism, the TCA cycle, and electron transport chain), FA synthesis and desaturation (ACLY, ACC, FASN, and SCD1), FAO (CPT1A), triglyceride synthesis and lipid-droplet formation (DGAT1/2), cholesterol uptake, trafficking, and esterification (LDLR, SOAT1, LXRβ, ABCA1, and V-ATPase), biotin-dependent metabolic regulation (SLC5A6 and HLCS), protein palmitoylation (DHHC enzymes), ferroptosis-associated antioxidant defenses (xCT and glutathione metabolism), and CD36-mediated signaling. Representative compounds are shown at their corresponding molecular targets. Red arrows indicate activation or metabolic flux, and the blunt-ended red lines indicate inhibition. The figure was created by the authors in https://BioRender.com. Repurposed drugs or experimental agents targeting these metabolic vulnerabilities and their stages of clinical development are summarized in Table 1.

Abbreviations: GLUT1: Glucose Transporter 1; HK2: Hexokinase 2; PEP: Phosphoenolpyruvate; PKM2: Pyruvate Kinase M2; PDK1: Pyruvate Dehydrogenase Kinase 1; ETC: Electron Transport Chain; TCA Cycle: Tricarboxylic Acid Cycle; α-KG: Alpha-Ketoglutarate; ACLY: ATP Citrate Lyase; ACC: Acetyl-CoA Carboxylase; CoA: Coenzyme A; CPT1: Carnitine Palmitoyltransferase 1; FASN: Fatty Acid Synthase; SCD1: Stearoyl-CoA Desaturase 1; MUFA: Monounsaturated Fatty Acid; DGAT1/2: Diacylglycerol Acyltransferase 1 and 2; SOAT1: Sterol O-Acyltransferase 1; LDLR: Low-Density Lipoprotein Receptor; LXRβ: Liver X Receptor Beta; ABCA1: ATP-Binding Cassette Transporter A1; HLCS: Holocarboxylase Synthetase; DHHC: Asp-His-His-Cys Palmitoyl Acyltransferase; XBP1: X-Box Binding Protein 1; SLC5A6: Solute Carrier Family 5 Member 6 (Sodium-Dependent Multivitamin Transporter); xCT: Solute Carrier Family 7 Member 11; System Xc^−^: Cystine/Glutamate Antiporter; GSH: Glutathione; GPX4: Glutathione Peroxidase 4; CD36: Cluster of Differentiation 36; V-ATPase: Vacuolar H+-ATPase; AEO: Anhydrous Enol-Oxaloacetate.

As shown in Figure 3 and summarized in Table 1, many of these metabolism targeting agents that have reached clinical evaluation are repurposed drugs with established safety profiles; however, the majority of newly developed metabolism-directed therapies remain in pre-clinical development. This trend highlights the translational advantages of drug repurposing and the growing interest in targeting metabolism as a therapeutic vulnerability in GBM.

## 8. Limitation

One of the major challenges in translating metabolic therapies to GBM is the heterogeneity of the disease. The differences in molecular subtypes, metabolic phenotype, treatment history, and immune composition can influence the extent to which an individual tumor depends on FA metabolic pathways [5,6,94]. Therefore, metabolic vulnerabilities in experimental settings may not be a uniform representation across all patients. This highlights the need for a more comprehensive understanding of metabolic diversity in GBM tumors. Although there have been advancements in understanding FA metabolism in GBM, several important questions remain regarding the cross talk between tumor progression, immune regulation, and therapeutic response. Current studies indicate that FA metabolism supports GBM growth, metabolic adaptation, and survival [8,9,22,23,42]. Similarly, Tregs use lipid-dependent metabolic pathways to maintain suppressive activity within metabolically hostile tumor microenvironments [10,16,17,60]. These observations suggest that FA metabolism may have a shared metabolic axis linking tumor fitness and immune suppression in GBM. However, much of the current understanding of these pathways is derived from pre-clinical studies, including cell culture, animal models, and mechanistic investigations. Although these studies have provided valuable insights into the biological significance of FA metabolism, clinical validation remains limited. Precise metabolic interactions connecting tumor cells, Tregs, and therapeutic response within the human GBM microenvironment require further investigation.

## 9. Conclusions

FA metabolism has been viewed as a pathway that supports the energetic and biosynthetic demands of GBM. However, the evidence-based studies reviewed here suggest a broader role for lipid metabolism within the GBM microenvironment. In addition to supporting tumor growth and metabolic adaptation, FA metabolism contributes to the suppressive activity of Tregs cells, which is involved in immune evasion and poor responses to ICIs. This evidence suggests that FA metabolism represents a common metabolic dependency shared by both GBM cells and Tregs. This overlap raises the possibility that lipid metabolic pathways contribute to the crosstalk between tumor progression and immune suppression. Therefore, in this review we bring together evidence from recent studies on GBM metabolism, Treg cell biology, and effects of Treg on ICIs to highlight a potential role for FA metabolism at the intersection of tumor progression and immune suppression. Although these areas have been investigated independently, the relationship between these pathways remains underexplored. Continued investigation of this intersection may further refine our understanding of the GBM microenvironment and immune evasion and inform future therapeutic strategies.

## Figures and Tables

**Table 1:** Metabolic pathway targeting agents investigated in glioblastoma.

Metabolic Drug	Effects on GBM Models	Clinical Development Stage
Metformin [120,121,122]	Suppresses GBM growth primarily by inhibiting mitochondrial complex I, leading to reduced ATP production and cellular energetic stress. This phenomenon tends to increase AMPK activation and subsequently suppresses mTOR signaling.	In phase II, in South Korea and Canada, for GBM treatment in combination with TMZ and radiotherapy.
DMAMCL/ACT001 [123,124,125]	Suppresses GBM proliferation by promoting PKM2 tetramerization which rewires aerobic glycolysis, resulting in reduced lactate-associated metabolic activity.	In phase I, in China and Australia, for GBM treatment.
Sulfasalazine [108,109]	Inhibits xCT, reducing glutathione synthesis and increasing GBM cell sensitivity to temozolomide.	In phase I, in Norway, for recurrent GBM treatment.
Ketoconazole [126]	Disrupts HK2-associated glycolytic metabolism and reduces glucose utilization and energetic support in GBM cells.	In phase I trial.
Posaconazole [127]	Disrupts HK2-associated glycolytic metabolism and reduces glucose utilization and energetic support in GBM cells.	In phase I trial.
Dichloroacetate (DCA) [128,129]	Inhibits Pyruvate dehydrogenase kinase 1, restoring PDH activity, and promotes pyruvate entry into mitochondrial oxidative metabolism instead of lactate-producing glycolysis.	In phase I trial.
Anhydrous Enol-Oxaloacetate (AEO) [130]	Acts as an oxaloacetate prodrug that promotes mitochondrial oxidative metabolism and reduces Warburg-like glycolysis and lactate production in GBM cells.	In phase II trial.
TVB-2640 [131,132]	Inhibit FASN enzyme, which disrupts tumor lipid biosynthesis and causes metabolic stress in GBM.	In phase II trial in combination with Bevacizumab
Ritonavir and Indinavir [133]	Reduces glucose uptake and lactate output, thereby restricting GBM cell growth.	Pre-clinical
WZB117 [134]	Inhibits GLUT1 activity and diminishes the self-renewal capacity of glioma stem-like cells.	Pre-clinical
WP1234 [135]	Suppresses glycolysis, resulting in decreased viability of GBM cells.	Pre-clinical
Phenformin [136]	Blocks mitochondrial complex I and reduce stemness-associated and mesenchymal gene expression in GSCs, leading to reduced tumor growth and longer survival in orthotopic models.	Pre-clinical
Mdivi-1 [137]	Inhibits Drp1-mediated mitochondrial fission and reduces proliferation of glioma stem-like cells.	Pre-clinical
LXR-623 [138]	Suppresses GBM growth by activating LXRβ-dependent cholesterol efflux pathways.	Pre-clinical
Archazolid B [139]	Disrupts cholesterol homeostasis in GBM cells by inhibiting vacuolar H+-ATPase (V-ATPase), causing lysosomal dysfunction and impaired intracellular cholesterol trafficking.	Pre-clinical
C75 [140]	Inhibits FASN, blocking de novo FA synthesis in glioma cells.	Pre-clinical
Avasimibe [35]	Inhibits SOAT1-mediated cholesterol esterification, resulting in ER cholesterol accumulation and downstream lipogenesis in GBM cells.	Pre-clinical
A-922500 [141]	Inhibits DGAT1-mediated triglyceride synthesis and lipid-droplet formation in GBM cells.	Pre-clinical
A939572 [47]	Inhibits Stearoyl-CoA Desaturase 1 (SCD1) to sensitize resistant glioma cells.	Pre-clinical
2-Bromopalmitate (2-BP) [142]	Inhibits Protein palmitoylation and disrupts XBP1-mediated ER stress adaptation in GBM cells.	Pre-clinical
Vstat120 [143]	Vstat120 activates CD36 signaling-dependent anti-angiogenic signaling and suppress blood vessel formation and glioma growth.	Pre-clinical
SB204990 [54]	Inhibits ACLY-dependent acetyl-CoA and suppresses FAO-associated radioresistance and CD47-mediated immune evasion in GBM.	Pre-clinical
Erastin [110]	Sensitizes GBM cells to temozolomide by inhibiting xCT-mediated cystine uptake, reducing glutathione-dependent antioxidant defense and promoting ferroptotic oxidative stress.	In vitro
Sulconazole [144]	Disrupts HLCS-mediated biotin distribution to biotin-dependent carboxylases and impair cholesterol metabolism.	In vitro
BMS303141 [145]	Suppresses GBM cell adhesion and migration by inhibiting ATP-citrate lyase (ACLY)-mediated acetyl-CoA production.	In vitro
Hydroxycitrate [146]	Inhibits ACLY-mediated acetyl-CoA production, suppressing glycolytic function and lipid biosynthesis.	In vitro
RSL3 [147]	Induces ferroptosis by inhibiting GPX4-mediated detoxification of lipid peroxides.	In vitro
PF-06424439 [148]	Lowers triglyceride synthesis.	In vitro
CAY10566 [41]	Inhibits SCD1-mediated FA desaturation and induce ER-stress-associated death in GBM stem cells.	In vitro

## Data Availability

No data were created or analyzed in this study. Data sharing is not applicable to this article.

## Abbreviations

ABCA1	ATP-binding cassette transporter A1
ACC	acetyl-CoA carboxylase
ACC1	acetyl-CoA carboxylase 1
ACC2	acetyl-CoA carboxylase 2
ACAD9	acyl-CoA dehydrogenase family member 9
ACLY	ATP citrate lyase
AEO	anhydrous enol-oxaloacetate
AIDS	acquired immunodeficiency syndrome
AKT	protein kinase B
AMPK	AMP-activated protein kinase
APC	antigen-presenting cell
ATP	adenosine triphosphate
ATGL	adipose triglyceride lipase
BBB	blood–brain barrier
CCL2	C-C motif chemokine ligand 2
CCL20	C-C motif chemokine ligand 20
CCL22	C-C motif chemokine ligand 22
CCL28	C-C motif chemokine ligand 28
CD25	cluster of differentiation 25
CD36	cluster of differentiation 36
CD47	cluster of differentiation 47
CNS	central nervous system
CoA	coenzyme A
CPT1	carnitine palmitoyltransferase 1
CPT1A	carnitine palmitoyltransferase 1A
CPT2	carnitine palmitoyltransferase 2
CTLA-4	cytotoxic T-lymphocyte-associated protein 4
CyTOF	cytometry by time of flight
DGAT	diacylglycerol O-acyltransferase
DGAT1	diacylglycerol acyltransferase 1
DGAT2	diacylglycerol acyltransferase 2
ER	endoplasmic reticulum
ETC	electron transport chain
FA	fatty acid
FABP5/7	fatty acid-binding proteins 5 and 7
FAO	fatty acid oxidation
FDA	U.S. Food and Drug Administration
FDG-PET	fluorodeoxyglucose positron emission tomography
Foxp3	forkhead box P3
FASN	fatty acid synthase
GBM	glioblastoma
GPR109A	G protein-coupled receptor 109A
GPX4	glutathione peroxidase 4
GSH	glutathione
GZMB	granzyme B
HIF-1α	hypoxia-inducible factor-1 alpha
HK2	hexokinase 2
HLCS	holocarboxylase synthetase
ICIs	immune checkpoint inhibitors
IDO	indoleamine 2,3-dioxygenase
IFN-γ	interferon-gamma
IL-2	interleukin-2
IL-10	interleukin-10
ITIM	immunoreceptor tyrosine-based inhibitory motif
ITSM	immunoreceptor tyrosine-based switch motif
LD	lipid droplet
LDH	lactate dehydrogenase
LDLR	low-density lipoprotein receptor
LKB1	liver kinase B1
LXRβ	liver X receptor beta
MAPK	mitogen-activated protein kinase
MCT1	monocarboxylate transporter 1
MHC II	major histocompatibility complex class II
MRI	magnetic resonance imaging
MSI-high	microsatellite instability-high
mTOR	mammalian target of rapamycin
MUFA	monounsaturated fatty acid
NAD^+^	nicotinamide adenine dinucleotide
NADPH	nicotinamide adenine dinucleotide phosphate
OXPHOS	oxidative phosphorylation
PD-1	programmed cell death protein 1
PD-L1	programmed death-ligand 1
PDH	pyruvate dehydrogenase
PDK1	pyruvate dehydrogenase kinase 1
PEP	phosphoenolpyruvate
PHD	prolyl hydroxylase domain
PI3K	phosphoinositide 3-kinase
PKM2	pyruvate kinase M2
PPAR-β	peroxisome proliferator-activated receptor beta
PTEN	phosphatase and tensin homolog
RR	radioresistant
SCAP	SREBP cleavage-activating protein
SCD	stearoyl-CoA desaturase
SCD1	stearoyl-CoA desaturase 1
SHP-2	Src homology region 2 domain-containing phosphatase 2
SLC5A6	solute carrier family 5 member 6
SOAT	sterol O-acyltransferase
SOAT1	sterol O-acyltransferase 1
SREBP-1	sterol regulatory element-binding protein 1
System Xc^−^	cystine/glutamate antiporter
TCA	tricarboxylic acid
TCGA	The Cancer Genome Atlas
Teffs	effector T cells
TERT	telomerase reverse transcriptase
TGF-β	transforming growth factor-beta
Th1	T helper 1
Th17	T helper 17
TILs	tumor-infiltrating lymphocytes
TME	tumor microenvironment
TMZ	temozolomide
Tregs	regulatory T cells
V-ATPase	vacuolar H^+^-ATPase
VEGF-A	vascular endothelial growth factor A
VHL	von Hippel–Lindau
WHO	World Health Organization
XBP1	X-box binding protein 1
xCT	solute carrier family 7 member 11
α-KG	alpha-ketoglutarate (2-oxoglutarate)
